# Scintillating Starbursts: Concentric Star Polygons Induce Illusory Ray Patterns

**DOI:** 10.1177/20416695211018720

**Published:** 2021-06-29

**Authors:** Michael W. Karlovich, Pascal Wallisch

**Affiliations:** Recursia Studios, New York City, New York, United States; Department of Psychology, New York University, New York City, New York, United States

**Keywords:** illusion, illusory contours, moiré pattern, Gestalt, unconscious inference, pincushion grid illusion, scintillating grid

## Abstract

Here, we introduce and explore *Scintillating Starbursts*, a stimulus type made up of concentric star polygons that induce illusory scintillating rays or beams. We test experimentally which factors, such as contrast and number of vertices, modulate how observers experience this stimulus class. We explain how the illusion arises from the interplay of known visual processes, specifically central versus peripheral vision, and interpret the phenomenology evoked by these patterns. We discuss how Starbursts differ from similar and related visual illusions such as illusory contours, grid illusions such as the pincushion grid illusion as well as moiré patterns.

Illusions have played a key role in understanding the principles of perceptual processing (Purkinje, 1825; van Buren & Scholl, 2018; Shapiro & Todorovic, 2017; Todorović, 2018), although this notion is not entirely without detractors (Braddick, 2018; Rogers, 2019). One important reason why studying these illusions can be helpful to understand visual processing is that they allow us to distinguish the mere sensation of physical object properties from the perceptual experience. This is perhaps clearest in the case of illusory contours. If contours are defined by a sharp change in luminance, it is hard to tell whether the observer directly perceives objects in the environment as they are or if the percept is constructed in the mind of the observer, because the output of a photometer and perceptual judgments agree (Fodor & Pylyshyn, 1981; Gibson, 1978). Conversely, illusory contours are not defined by changes in luminance and would be invisible to a photometer, whereas human observers readily perceive them. This situation is mirrored by the response properties of neurons in primate visual cortex—neurons in area V1 respond to luminance-defined, but not illusory contours (von der Heydt & Peterhans, 1989; von der Heydt et al.,1984). A classic example of illusory contours is Kanizsa’s triangle, which most observers perceive as a bright triangle occluding three black circles—as opposed to the three black *Pac-Man*-like shapes that are actually defined by luminance (Kanizsa, 1955). Kanizsa’s triangle also illustrates a different principle, by which organisms interpret scenes in ways that require the fewest assumptions, or are probabilistically most plausible (Koffka, 1935). This interpretation is consistent with the position of the *unbewusster Schluss* or unconscious inference (von Helmholtz, 1867); as stimulus configurations are often ambiguous, the brain is using probabilistic inference to parse an image (Pitkow & Angelaki, 2017; Stocker & Simoncelli, 2006).

Here, we introduce a related, but unique type of visual stimulus that evokes illusory rays that seem to shimmer or scintillate (see Figure 1). We call this stimulus the *Scintillating Starburst*, motivated by the fact that the illusory percept closely resembles a visual pattern what is—in graphic design—referred to as a *starburst*.

**Figure 1.**
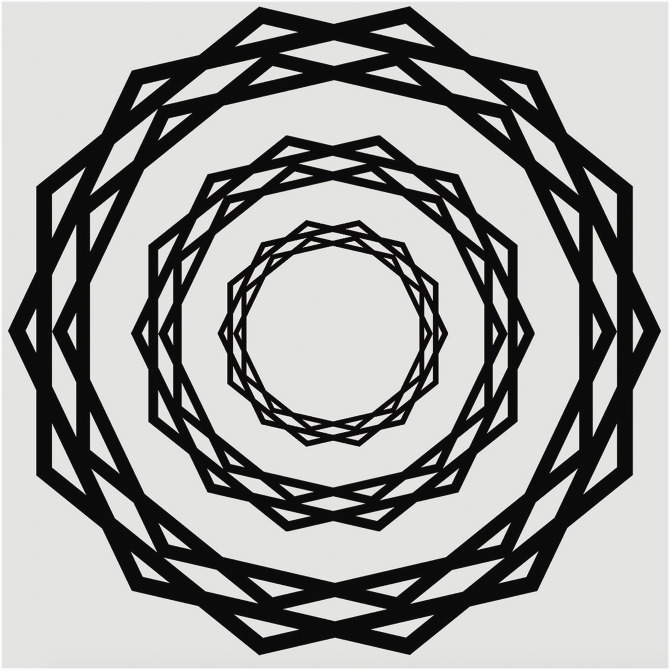
The *Scintillating Starburst* stimulus. This stimulus is made up of several star polygons with faces that bisect each other (see the Method section for details). Most observers perceive fleeting rays, beams, or lines emanating from the center that appear to be brighter than the background.

We are aware that this stimulus appears superficially similar to a number of previously described effects. However, it differs from these in important and specific ways:

1. The pincushion grid illusion

This is a grid-based illusion that evokes fleeting and faint illusory lines connecting the points of the grid diagonally (Schachar, 1976); the appearance of the illusion strengthens if the checkerboard lattice is color-enhanced (Roncato et al., 2016). There are four differences to Scintillating Starbursts.

- The pincushion grid illusion is evoked by simple lattices (see Figure 3E), where each image element is identical and repeating. Scintillating Starbursts are defined by scaled pairs of concentric star polygons.
- In the pincushion grid illusion, the illusory lines are crisscrossing the lattice diagonally, that is, if the lattice is horizontal and vertical, the illusory lines are oblique, and if the lattice is oriented obliquely, the illusory lines connect horizontally and vertically. In the Scintillating Starbursts, this is not the case. Instead, the rays connect the center of the pattern directly to the intersection points of the star polygons. In other words, the relation between luminance-defined and illusory percepts is direct, not oblique.
- The fleeting streaks in the pincushion grid illusion seem to be of extremely high spatial frequency, whereas the rays in the Scintillating Starbursts seem to be considerably thicker and scale as a function of the properties of the wreaths (see the Method section).
- Curiously, the illusory percept from Scintillating Starbursts is predicted by the Fourier transform (FT) of the stimulus, whereas this is not the case for the pincushion grid illusion. Vision scientists were taken by the observation that neurons in early visual cortex are tuned for spatial frequency (Enroth-Cugell & Robson, 1966), suggesting that the visual system might effectively perform a linear systems analysis on the visual image (Campbell & Robson, 1968), although this view has been somewhat tempered as of late (Carandini et al., 2005). Schachar (1976) used the pincushion grid illusion to demonstrate that there is no clear correspondence between percept and FT, as the FT of the grid in the pincushion grid illusion has no diagonal components. This view was challenged by Rudee et al. (1977). Ochs (1979) argues that the brain might not use these Fourier components, even if they were present in the stimulus. Morgan and Hotopf (1989) note that there are pincushion-style grids that evoke percepts that do not correspond to Fourier components. Conversely, in Scintillating Starbursts, the match between perception and FT is striking, particularly for those made up of odd-sided polygons (see Figure 2), as in these cases, the FT directly corresponds to the percepts. In even-sided polygons, only the number of spokes matches the FT, whereas their orientation is offset by a quarter of the central angle of the polygon.

**Figure 2.**
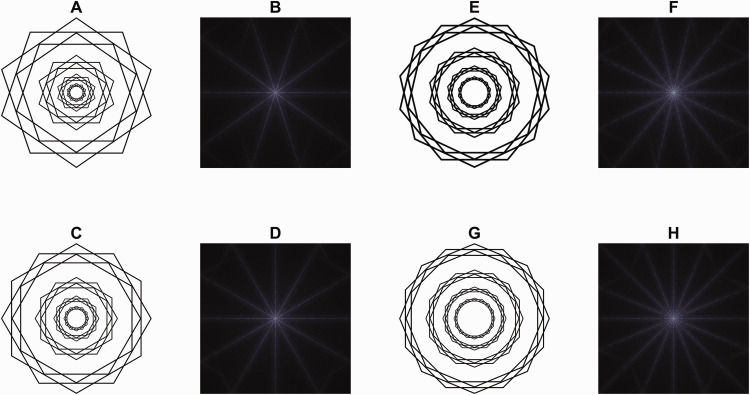
Starbursts and their FT. A: {10/2} Scintillating Starburst. The 10 perceptual rays match the components of the FT in B. C: {12/2} Scintillating Starburst. The 12 perceptual rays match the components of the FT in D. E: {14/2} Scintillating Starburst. The 14 perceptual rays match the components of the FT in F. G: {16/2} Scintillating Starburst. The 16 perceptual rays match the components of the FT in H. {p/q} denotes Schläfli notation, where p is the number of vertices and q is the turning number (Coxeter, 1974, p. 14).

To our knowledge, this behavior—where the illusory percept matches the FT of the stimulus—has not been previously described; see Figure 3. Admittedly, this correspondence might be incidental, but it is curious enough to be worth noting.

**Figure 3.**
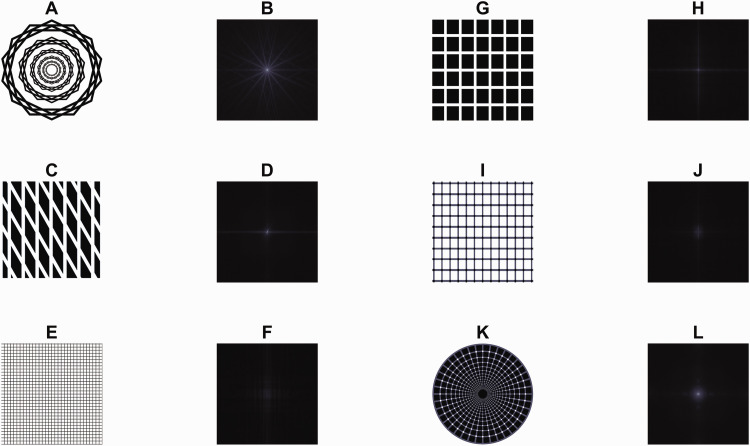
FT of Starbursts and seemingly similar phenomena. A: A Scintillating Starburst stimulus with associated FT in B. C: Phantom bands of a Motokawa grid with associated FT in D. E: The Pincushion grid illusion with associated FT in F. G: The Hermann grid with associated FT in H. I: A scintillating grid with associated FT in J. K: A radial scintillating grid with associate FT in L.

2. Phantom bands and moiré patterns

A similar effect from grid-like lattices has been described as ‘phantom bands’ (Motokawa, 1950). Briefly, a rhombic alignment of the lattice seems to abolish the dark spots at the intersection points of the Hermann grid; instead, dark phantom bands seem to appear (Hamburger et al., 2012). It is possible that Scintillating Starbursts can be conceived of as phantom bands, but there are several differences between phantom bands and the illusory rays in Scintillating Starbursts. First, like in the pincushion grid illusion, there are no components in the FT of the rhombic grid lattice that correspond to the phantom bands (Figure 3D), unlike for Scintillating Starbursts. Second, in Scintillating Starbursts, the wreaths are separated by a substantial portion of background. Conversely, in a rhombic lattice, a low-pass filtered version of the stimulus would contain luminance-defined phantom bands, whereas a low-pass filtered version of Scintillating Starbursts does not contain rays.

Similarly—and more generally—Moiré patterns are interference patterns that result from regular striped gratings being overlaid with similar patterns with some—often oblique—offset (Oster, 1968). Although the phenomenological appearance of these patterns can be striking, the beat patterns are actually present physically; in other words, they are luminance-defined patterns, unlike in Scintillating Starbursts.

3. Other scintillating illusions

Superficially, the Scintillating Starburst shares features of similar phenomena such as the Hermann grid (Hermann, 1870), scintillating grid (Schrauf et al., 1997), or the radial version of the scintillating grid (Figure 3G to K), but none of these have Fourier components that align with the percept.

4. The Spokes illusion

Finally, the rays or beams observed to connect opposing bisection points of the polygons are reminiscent of the ‘spokes’ illusion (Holcombe et al., 1999). In this illusion, perceived spokes emerge when fixating in the middle of revolving high-contrast objects. Although it is the case that the rays stabilize and strengthen when the polygons of the Starburst stimulus are rotating, the spokes illusion is fundamentally a motion illusion, whereas motion is not necessary in the Starburst. Moreover, the spokes do not seem to appear immediately, yet the rays in the Scintillating Starburst appear to emerge instantly, and without a need to fixate in the center.

Thus, we consider Scintillating Starbursts sufficiently novel and interesting to explore the space of stimulus parameters that modulate subjective experiences of ray strength (RS) and quantify their relative impact on phenomenology.

## Method

We were interested in investigating what modulates the visual experience of Scintillating Starbursts. To do so, we employed the empirical paradigm detailed later.

### Stimuli

**Figure 4.**
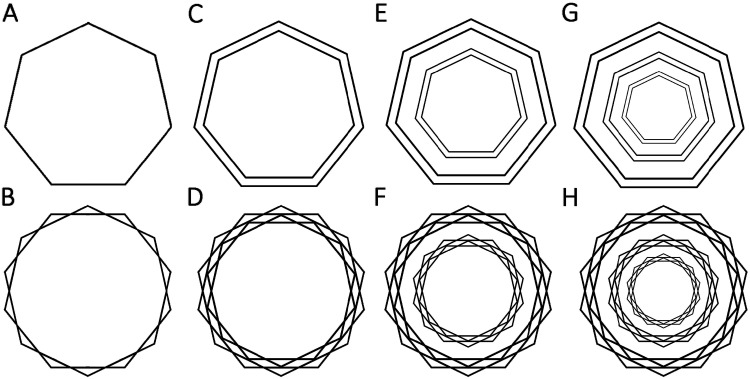
An illustration of how the Scintillating Starburst stimulus was constructed. Top row: nonbisecting stimuli, bottom row: bisecting stimuli. A: A single polygon—here, a heptagon, which we call a strand. B: Two heptagons with faces that bisect each other to form a {14/2} star polygon known as a tetradecagram, which we call a braid. C: Two concentric nonbisecting strands. D: Two braids scaled so they just touch, making up one wreath. E: Two concentric nonbisecting pairs of strands. F: Two concentric wreaths. G: Three concentric nonbisecting pairs of strands. H: Three concentric wreaths. This stimulus corresponds to the *Scintillating Starburst stimulus* shown in Figure 1, but on a white background and smaller.

The *Scintillating Starburst* stimulus seen in Figure 1 elicits transient shimmering rays that seem to be emanating from the center for most observers. This stimulus was designed in the following way. We used MATLAB (Mathworks, Inc., Sherborn, MA) to construct a regular heptagon (central angle = 51.143 degrees, rounded to 3 decimals), assuming a line width of 2 points (see Figure 4A). Figure 4B was created by adding a second, identical heptagon rotated by 25.71 degrees (π/7 radians) so that the faces of the two heptagons bisect each other forming a {14/2} star polygon known as a regular tetradecagram (Coxeter, 1973). We call the image element depicted in Figure 4B a braid. Figure 4C was created by adding a scaled version (89.95%) of the heptagon in Figure 4A to itself. Figure 4D was created by adding a second, scaled braid. The scale factor was chosen so that the two braids just touch each other; in other words, the vertices of the smaller tetradecagram bisect the midpoints of the larger tetradecagram, forming a wreath. Figure 4C depicts a pair of strands whereas Figure 4D depicts a wreath. The image in Figure 4E was created by adding another pair of pair of strands to 4C such that the second pair is a scaled-down version (66%). The image in 4F was similarly created by adding another wreath to 4D, using the same scale factor. Thus, 4E and 4F feature two non-bisecting pairs of strands and wreaths each, respectively. Finally, Figure 4G and H was created by adding an additional pair of strands or wreath using the same scale factor so that these images are featuring three pairs of strands and wreaths, respectively. One could repeat this principle to create stimuli with even more wreaths. One could also vary the number of vertices in the polygon.

In fact, we did just that to parametrically create visual stimuli varying in five stimulus dimensions: (1) number of vertices of the polygon (3 levels: 3 5 7), (2) contrast (3 levels: 0.1 0.55 1), (3) line width of the innermost wreath (3 levels: 0.5 1 1.5 points), (4) number of wreaths (3 levels: 2 4 6), and (5) whether the braids of the wreaths bisect each other (2 levels: yes and no).

Fully crossing these stimulus dimensions yields the set of 162 unique stimuli we used in this study. All other stimulus dimensions were kept the same across all stimuli so as to not further increase the number of stimuli in the set. All stimuli were presented on a white background at a size of 600 × 600 pixels, delivered remotely.

**Figure 5.**
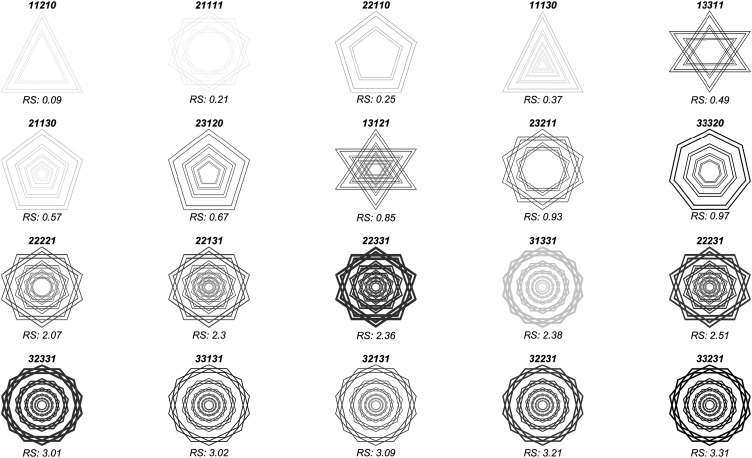
A representative sample of 20 out of the 162 stimuli used in this study. Pictograms of the stimuli are arranged in increasing order of average ray strength reported by our sample of observers. Top rows: weak ray strengths. These rows are predominantly made up of triangular polygons, low-contrast stimuli, and nonbisecting stimuli. Second to last row: stimuli with moderate experienced ray strength—predominantly bisecting pentagons. Bottom row: These five stimuli evoked the strongest ray experiences. These stimuli consist of high-contrast bisecting heptagons with several wreaths. This stimulus is made up of several interlocking polygons that bisect each other’s faces (see the Method section for details). Most observers perceive fleeting rays, beams, or lines emanating from the center that appear to be brighter than the background. The label above each icon indicates the level of the independent variable, in order (number of vertices per polygon, contrast, line width, number of wreaths, and whether the polygon faces bisect each other). The RS value below each icon denotes the average experienced ray strength.

See Figure 5 for an illustration of a representative sampling of this space as well as the average experienced RS evoked by these stimuli in our sample of observers. As you can see, our set of stimuli evoked a wide range of responses and was effective in modulating the experience of the observers. The reason we picked these stimulus dimensions and not others is to avoid the curse of dimensionality—there is a vast space of visual parameters to vary, many of which have no impact on the effect. For instance, extensive piloting suggested that color, size, or uncoiling the wreaths to yield nonradial ray patterns did not seem to affect experienced RS much.

### Participants

We recorded data from a total of 122 participants. We recruited naive observers from the New York University student community and the general public. We excluded data from eight participants who completed the study implausibly fast (less than 2 seconds per question, median response time per question = 6.08 seconds), six participants who reported that they did not respond seriously, and from 1 participant with more than three missing responses. Importantly, we decided on these exclusion criteria before looking at any of the results. Thus, we retain data from seven-eigths of the initial sample for further analysis. In this sample, the median age was 19.5 years. A total of 66 participants identified as female, 40 as male, and 1 participant did not disclose a gender. We consider this sample to be sufficiently large for this study to be adequately powered (Wallisch, 2015).

### Task

All observers were asked to answer a number of questions about how strongly they experience the RS of these stimuli. Response options were as follows: “I do not see any bright lines, rays, or beams,” “I maybe see bright lines, rays, or beams, but they are barely noticeable,” “I see bright lines, rays, or beams, but they are subtle and weak,” “I clearly see bright lines, rays, or beams,” and “I see strikingly bright lines, rays, or beams”. We took these response options to form an intuitive 5-point Likert scale and scored them as 0 to 4 in terms of RS, respectively. All of these stimuli were presented in random order.

This study was hosted online (Surveymonkey.com), and all procedures were approved by the New York University Institutional Review Board, the University Committee on Activities Involving Human Subjects (UCAIHS).

### Data Analysis

All data were analyzed with MATLAB (Mathworks, Inc., Sherborn, MA). Specifically, we used a 3 × 3 × 3 × 3 × 2 repeated measures analysis of variance (ANOVA) to analyze the data on the experienced RS of the 162 unique stimulus patterns. As our study is adequately powered, we set the significance threshold at .005, to avoid false positives (Benjamin et al., 2018).

## Results

Using the methods described earlier, we attempted to answer the following questions:

### How Do Variations of Stimulus Parameters in Different Dimensions Affect the Perceived RS of Observers When Looking at Scintillating Starburst Stimuli?

To answer this question, we performed a five-way repeated measures ANOVA on the responses to the 162 stimuli. See Table 1 for results.

**Table 1.** Repeated Measures ANOVA Table.

Source	Sum of squares	*df*	Mean squares	*F*	*p*
Intercept	13,046.79	1	13,046.79	283.74	**4.000e-29**
NV	947.23	2	473.62	107.96	**3.261e-31**
CT	1,010.69	2	505.34	182.22	**2.087e-43**
BL	174.87	2	87.43	147.80	**3.073e-38**
NW	2,871.15	2	1,435.58	413.42	**7.873e-67**
BS	2,687.72	1	2,687.72	258.91	**8.252e-28**
NV × CT	82.04	4	20.51	36.36	**2.137e-25**
NV × BL	11.99	4	3.00	9.09	**5.375e-07**
CT × BL	2.71	4	0.68	1.66	0.158
NV × NW	159.56	4	39.89	44.33	**4.517e-30**
CT × NW	89.27	4	22.32	43.29	**1.791e-29**
BL × NW	27.14	4	6.78	15.00	**2.556e-11**
NV × BS	414.62	2	207.31	110.66	**9.831e-32**
CT × BS	240.20	2	120.10	150.06	**1.336e-38**
BL × BS	76.99	2	38.50	84.12	**2.756e-26**
NW × BS	544.35	2	272.17	107.29	**4.386e-31**
NV × CT × BL	6.39	8	0.80	2.58	0.009
NV × CT × NW	9.73	8	1.22	3.69	**3.140e-04**
NV × BL × NW	4.05	8	0.51	1.58	0.127
CT × BL × NW	27.70	8	3.46	10.58	**4.363e-14**
NV × CT × BS	51.03	4	12.76	29.20	**6.047e-21**
NV × BL × BS	13.29	4	3.32	9.13	**5.067e-07**
CT × BL × BS	9.49	4	2.37	6.86	**2.507e-05**
NV × NW × BS	46.67	4	11.67	18.88	**4.609e-14**
CT × NW × BS	22.79	4	5.70	11.69	**6.419e-09**
BL × NW × BS	28.06	4	7.01	14.30	**8.137e-11**
NV × CT × BL × NW	8.61	16	0.54	1.70	0.041
NV × CT × BL × BS	3.92	8	0.49	1.58	0.126
NV × CT × NW × BS	5.35	8	0.67	1.94	0.052
NV × BL × NW × BS	5.15	8	0.64	1.76	0.081
CT × BL × NW × BS	31.48	8	3.94	12.35	**1.280E-16**
NV × CT × BL × NW × BS	8.70	16	0.54	1.60	0.062
Error	4,000.37	87	45.98	1.00	0.500

*Note*. Factors: NV = number of vertices of the polygons; CT = contrast; BL = base line width; NW = number of wreaths; BS = bisecting strands. Interactions are denoted with ×. Significant effects are bolded.

**Figure 6.**
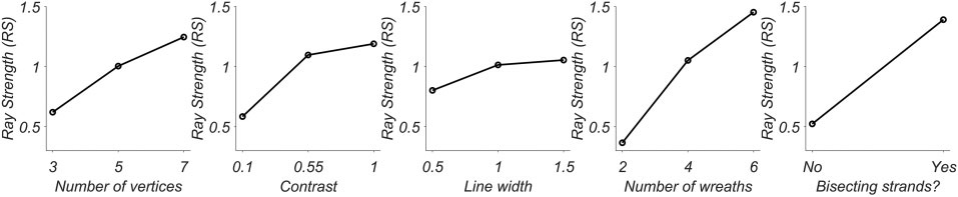
Means plot of main effects of the repeated measures ANOVA from Table 1. Each panel corresponds to a different independent variable (levels are on the *x* axis), whereas the *y* axis represents the average experienced ray strength (RS) reported by our observers.

All five main effects were significant, with variable modulation range; see Figure 6 for an illustration of the means plots of the main effects.

**Figure 7.**
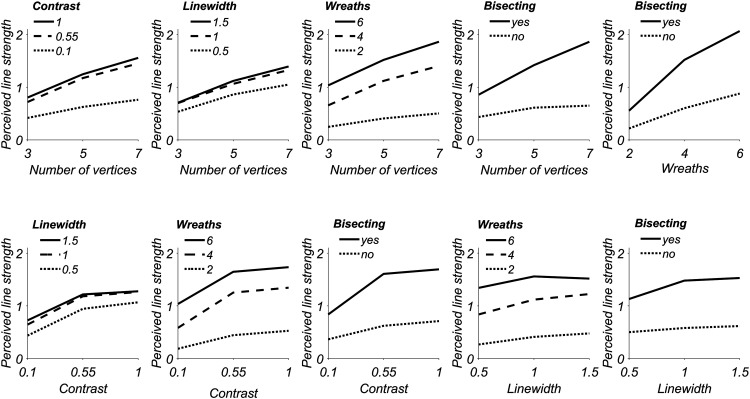
Two-way interaction plots between all independent variables used in the repeated measures ANOVA. Each panel corresponds to a different independent variable (levels are on the *x* axis), whereas the *y* axis represents the average experienced ray strength (RS) reported by our observers. Each line corresponds to a different level of the second independent variable, as detailed in the legends.

The number of wreaths modulates the experienced RS most strongly, whereas the modulation from changing the base line width is only modest. However, note that—by themselves—none of these parameters elicit clear or strong ray experiences. This suggests that the full experience of these rays stems from a combination of factors. See Figure 7 for an illustration of all two-way interactions. All two-way interactions with the exception of the interaction between contrast and linewidth are significant.

Even the strongest two-way combinations, that is, many bisecting polygons or many vertices plus many wreaths by themselves only elicit weak responses, on average. Most three-way interactions and even one four-way interaction (that between contrast, line width, number of wreaths, and whether the strands bisect) are significant. Although it is difficult to meaningfully interpret higher order interactions, we believe that this pattern of results suggests both that the modulation range of perceptual experiences as a function of stimulus parameters is large and that a strong ray experience relies on the optimal combination of many stimulus dimensions at once.

**Figure 8.**
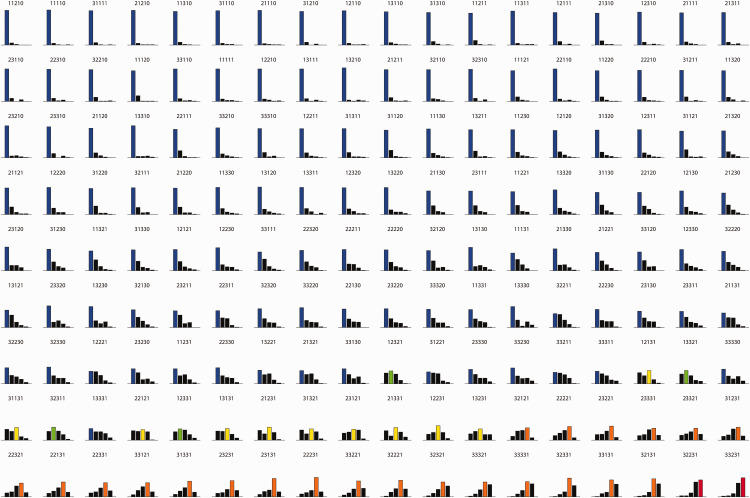
Ray strength responses evoked by all 162 stimuli in our set, arranged by increasing average ray strengths. The label above each histogram denotes the combination of independent variable levels that corresponds to the stimulus, as in Figure 3. The bars represent the response histogram evoked by each stimulus. The modal response is colored in terms of the following color code: blue: no rays, green: maybe rays, yellow: subtle rays, orange: clear rays, red: striking rays.

In addition, we suspect that combining individual responses by taking the mean does not fully represent the fine structure of responses evoked by these stimuli. Thus, in Figure 8, we present the stimulus space ordered by increasing mean response in terms of response histograms.

As expected on the basis of the ANOVA means plots, much of our stimulus space did not evoke strong responses in most of our observers. Only a few stimuli are experienced by most observers as exhibiting strikingly bright rays, when all parameters combine optimally in all of the dimensions we varied.

## Discussion

In this article, we explored a unique kind of stimulus that evokes ghostly or ephemeral illusory rays that appear to shimmer or scintillate. Curiously, the number and orientation of rays in the illusory percept closely corresponds to what would be predicted from an FT of the stimulus. We ascertained that the RS experienced by observers when viewing this stimulus type is modulated by all stimulus dimensions we suspected to be relevant when piloting the study, namely the number of vertices of the polygons, contrast, the line width of the wreaths, the number of wreaths, and whether the polygons are bisecting or not. The strongest effect was yielded by the number of wreaths, followed by whether the strands are bisecting, stimulus contrast, line width of the braids, and the number of vertices of the polygons, in that order. Interestingly, almost all two-way interactions of these stimulus dimensions, most three-way interactions, and even one four-way interaction (that between line width, contrast, number of wreaths, and whether the polygons are bisecting or not) were also significant. This is interesting, as no stimulus dimension by itself produces a strong effect, only the optimal confluence of many stimulus parameters does so. We believe that these results are consistent with probabilistic inference—for instance, the percept of illusory lines from an occluder is more likely if there are more intersection points where the vertices bisect, and if this happens at higher contrast. This is not implausible, as deciding on a coherent interpretation of ambiguous visual information is a fundamental challenge faced by the visual system. Of course, probability by itself is not sufficient—the specific stimulus situation matters—for instance, a row of street lights does not evoke the impression of a bright like that connects them. But in the case of street lights, the bright beacons are broken up by the darkness of the night. This darkness is unambiguously present. However, in the case of Starbursts, the bright beacons are separated by background of the same color, yielding the percept of an occluder of that color on top of the stimulus.

We are aware of several limitations of this research. First, this stimulus class seems to be modulated by many parameters over a wide range of values, which creates a combinatorial explosion. For instance, if we even just added uncoiled versions of the wreaths to the fully crossed design, observers would have been presented with twice the number of stimuli—324 trials. Moreover, using just 5 instead of 3 levels per parametric dimension would have yielded 1,250 distinct versions of these ray pattern stimuli, which is impractical to use in a single experiment. Thus, we are aware that we are undersampling the full stimulus space.

Second, the 0.1 contrast condition was probably too weak to be seen readily, regardless of the other stimulus dimensions, establishing a floor effect. In hindsight, we should have used logarithmic spacing of contrast levels—0.25, 0.5, and 1. However, because observers are unlikely to be using calibrated monitors in an online study anyway, this probably does not matter much as the visual effects of the Scintillating Starburst stimulus are fairly stable over a large parameter range.

Nevertheless, we believe that we have sufficient empirical evidence to elicit the key mechanism that underlies and brings about this phenomenon.

We attribute this effect to several known visual processes that work in conjunction to bring about the illusory scintillating rays, much like in the case of the *lilac chaser*, which is a combination of Troxler fading, after-images, and the phi effect (Bach, 2005).

**Figure 9.**
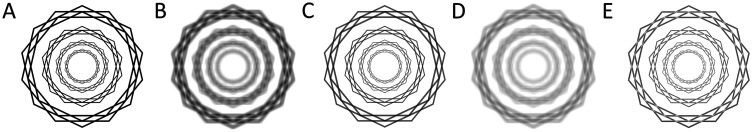
The suggested mechanism underlying the Scintillating Starburst effect. A: A Starburst made up of four concentric wreaths at high contrast. B: A low-passed version of this stimulus. As you can see, there are islands of relative brightness where the faces bisect. C: A version of the stimulus in A where the braids are made up of polygons at half-contrast. Only the bisection spots—where the polygons sum—are at full contrast. D: A low-pass filtered version of the stimulus in C. Note that there are no net bright spots in this blurry version, as the full contrast bisections blend in with the rest of the stimulus. E: This is the stimulus in C subtracted from the stimulus in A, akin to Movshon et al. (2003). As you can see, bright features emerge in this subtractive version. This stimulus appears to have even stronger rays, likely because the aligned bisection points are luminance-defined and objectively brighter than their surroundings, much like in a moiré pattern.

At the heart of our explanation of this effect is the different in spatial resolution between foveal and peripheral vision. This difference is seeded in the retina, with midget ganglion cells predominating in the fovea and parasol ganglion cells in the periphery (Masland, 2001; Rodieck & Rodieck, 1998). As a consequence, foveal vision provides a high-resolution image, whereas peripheral vision is blurry. This is important due to a feature of Scintillating Starburst stimuli (see Figure 9). The strands making up the braids of the Scintillating Starburst are at full contrast, yielding a compound stimulus of uniform luminance (Figure 9A). Adding up half-contrast strands yields a compound stimulus that is not of uniform luminance—the intersection points are darker than their surroundings—but is a strictly linear summation of the components (Figure 9C). This matters because parts of the visual system effectively perform a low-pass filter of the visual input (for instance, magnocellular neurons or neurons with receptive fields in the periphery). In the low-pass filtered version of the image, the regions where the strands that make up the braids intersect are therefore brighter if braids are made up of full contrast strands, as their linear summation is sublinear at the points of intersection (Figure 9B), whereas this is not the case for the half-contrast strands, as their summation is fully linear (Figure 9D). Consequently, the appearance of rays is diminished for half-contrast braids. This point is reinforced by the fact that the appearance of rays is enhanced if one subtracts the Starburst made up of half-contrast braids from the full-contrast ones (Figure 9E), revealing the existence of luminance-defined beacons. Similar observations pertaining to features defined by stimulus nonlinearities have been made elsewhere, for instance, regarding the perception of moving compound stimuli (Movshon et al., 2003). We would like to emphasize that this account is speculative in nature, as we did not investigate the effect of eccentricity or that of spatial filtering. We were unable to do so, as determining the mechanism was not the focus of our investigation. We aimed to map the phenomenology of this effect, and the stimulus space was already quite large. We think that—now that optimal stimulus conditions that drive the effect have been established—exploring the mechanism by systematically varying eccentricity and different levels of spatial filtering is exciting and apt avenues for future research.

Yet, it stands to reason that the most compelling interpretation of this unlikely spatial alignment of bright beacons (see Figure 9) is that there is an actual bright line that is occluding the black wreaths, just like in a classical illusory contour (e.g., Kanizsa’s triangle). Peripheral vision simply does not have the resolution to discern that the bisection points are not actually brighter (Otten et al., 2017) and notoriously conflates multiple objects that fall in the same large receptive field (Gurnsey & Biard, 2012; Gurnsey et al., 2011; Pelli & Tillman, 2008). We think that the reason the rays seems to sparkle—unlike classical illusory contours (Kanizsa, 1976)—is that central vision does have the resolution to tell that the luminance in the bisection centers is actually not brighter (Westheimer, 1982). This dynamic competition between foveal (“no bright line is occluding the wreaths”) and peripheral vision (“it is likely that there is a bright line occluding the wreaths”) renders them so fleeting, as human observers make frequent eye movements during visual inspection. Note that other scintillating stimuli are also believed to be due to dynamic competition of the underlying neural circuits (Pinna et al., 2002), echoing other illusions that rest on the interplay between foveal and peripheral vision (Shapiro et al., 2010). Moreover, if the stimulus falls in the far periphery, the rays disappear altogether, presumably because there is not enough resolution to resolve these beacons.

Thus, we conclude that Scintillating Starbursts are due to a compound illusion that combines several visual processes to bring about the effect, much like the Lilac chaser. Our theoretical interpretation integrates all of the empirical evidence presented in this article. We observed that the effect gets stronger if there are more wreaths and more vertices per polygon (although piloting suggests that if there are too many vertices in a polygon, the stimulus becomes too indistinguishable from a circle and the effect decreases again). This will increase the number of coincidental spatial alignments, thus provide stronger evidence for a more compelling interpretation—an occlusion by a bright streak that goes away when looking at it directly, because it is incompatible with the information provided by the fovea. We also observed that the effect gets stronger with increased line width and contrast, both of which would render the ghostly bright dot at the bisection points stronger. Finally, we observed that bisecting the polygons increases the effect, which makes sense as there are no—relatively—bright intersections without such overlapping polygons. The fact that this effect results from the confluence of many factors also likely explains why it has remained unnoticed or unreported for so long—the only way to get strong effects from ray patterns is for all of these factors to be optimal. If true, this could be considered another instance of a *crowd within* (Vul & Pashler, 2008)—here due to the interplay between peripheral and central vision.

The class of stimuli we termed *Scintillating Starbursts* which gives rise to this compound illusion opens several avenues for future research. First, one wonders at which level of the visual hierarchy this effect arises. If it is similar to classical illusory contours, we would expect physiological correlates as early as V2, but not V1 (von der Heydt, Peterhans, & Baumgartner), but it could manifest later, due to the interplay between foveal and peripheral vision which might happen at a subsequent stage of visual processing and awareness. Second, piloting suggests that subjective states such as sleep deprivation might modulate the strength of the effect substantially, but this needs to be systematically verified, and in a larger sample. Third, it would be useful to empirically study the larger parameter space that we did not explore here. We fixed all parameters other than the five dimensions we mentioned in this article at constant values (e.g. we used a turning number of 2), so as to not increase the number of stimuli beyond what is feasible to accomplish in one study. For instance, the braids that make up a wreath are scaled versions of each other. Small changes in this scale factor dramatically change the phenomenological appearance of the stimulus, but we kept this scale factor constant across all stimuli. Fourth, if our explanation of this mechanism is correct, changing the luminance of the bisection—and more generally intersection—points to compensate for the relative contrast in the low-pass filtered image due to nonsuperposition should abolish the rays. It would be interesting to know how much contrast one has to add to prevent scintillating rays, but this would require the use of calibrated monitors, so it is beyond the scope of online studies.

To summarize, we believe that both Scintillating Starbursts and the pincushion grid illusion (as well as possibly phantom bands) share the same root—or mechanism—nonlinear beacons in the periphery that are interpreted by illusory lines—but that a Scintillating Starburst is a much stronger implementation of this principle—in the pincushion grid illusion, the illusory lines are thin (if they are thicker, the grid itself starts to scintillate, perceptually) and nonrobust (there is a tendency to seem them mostly along the cardinals, see Movie 1), whereas in the Starburst configuration, the percept of the illusory lines is rotation invariant and scale invariant as well as more striking (see Movie Video 2).

Finally, we wonder about individual differences in the experienced RS between observers. For instance, there might be an individual threshold of evidence (the number of aligned intersection or bisection points) that a given observer needs to see to reliably perceive scintillating rays and their propensity to “connect the dots” (where coincidence is no longer subjectively consistent with parsimony). This might carry over to other domains, such as the perception of constellations or the belief in conspiracy theories.

**Movie 1.** Pincushion grid illusion.

**Movie 2.** The Scintillating Starburst stimulus.
